# An association of *ABCG8*: rs11887534 polymorphism and HDL-cholesterol response to statin treatment in the Polish population

**DOI:** 10.1007/s43440-021-00302-7

**Published:** 2021-06-26

**Authors:** A. Sałacka, A. Boroń, I. Gorący, I. Hornowska, K. Safranow, A. Ciechanowicz

**Affiliations:** 1 Department of Family Medicine, Pomeranian Medical University, Szczecin, Poland; 2 Department of Clinical and Molecular Biochemistry, Pomeranian Medical University, Szczecin, Poland; 3 Department of Biochemistry and Medical Chemistry, Pomeranian Medical University, Szczecin, Poland

**Keywords:** **S**tatin, Lipid response, *ABCG8*, Gene polymorphism

## Abstract

**Background:**

Variation in lipid changes in response to statin treatment is associated with genetic polymorphism. Sterolin-1, encoded by *ABCG5*, and sterolin-2, encoded by *ABCG8*, together form a sterol transporter. There are some reports indicating association of rs11887534 (*ABCG8*:c.55G > C) polymorphism with lipid concentrations, both prior to and after statin treatment. The aim of this study was to analyze both baseline plasma lipids and their concentrations in response to statin treatment with regard to *ABCG8*: rs11887534 polymorphism in Caucasian patients of Polish origin.

**Methods:**

The study group consisted of 170 consecutive adult out-patients treated with atorvastatin or simvastatin for a minimum of 2 months. Concentrations of triglycerides (TG), total cholesterol (TC), LDL-cholesterol (LDL-C) and HDL-cholesterol (HDL-C) were measured before and after statin treatment. The *ABCG8* polymorphism was identified by mini-sequencing genomic DNA extracted from peripheral blood leukocytes.

**Results:**

There were no significant differences in regard to *ABCG8* variants for baseline TG, TC, LDL-C and HDL-C as well as for TG, TC or LDL-C concentrations after statin treatment. However, patients carrying at least one C allele showed a decrease in post-statin HDL-C concentrations and the absolute and relative changes between post- and pre-statin HDL-C concentrations were negative in contrast to positive values in wild-type homozygotes.

**Conclusions:**

Our results suggest that the c.55C allele of the *ABCG8*: rs11887534 polymorphism might be associated with decrease in HDL-cholesterol in response to statin treatment in Polish patients.

## Introduction

Statins are competitive inhibitors of the 3–hydroxymethyl–3–methylglutaryl coenzyme A (HMG-CoA) reductase, which is a rate-limiting enzyme in cholesterol biosynthesis [1]. A decrease in cellular cholesterol concentrations caused by statins stimulates cell-surface expression of low-density lipoprotein (LDL) receptors on hepatocytes, which in turn increase the removal of circulating LDL cholesterol (LDL-C)[2]. Treatment with statins not only causes various degrees of LDL-C decrease but also a decrease in total cholesterol and triglyceride concentrations as well as a HDL-C increase [3, 4]. Hasvold et al. indicated that statin-induced changes in LDL-C and HDL-C are unrelated and many patients initiated on statins experience a paradoxical decrease in HDL-C [5]. There is also evidence that the inter-individual variability in lipid response to treatment with statins may be associated with genetic polymorphisms [1, 6–11].

The *ABCG8* gene is located on chromosome 2p21 in a head-to-head orientation with the *ABCG5* gene. *ABCG5* encodes sterolin-1 and *ABCG8* encodes sterolin-2. Both sterolins are non-functional half-transporters which have to form the heterodimer to gain sterol transport functionality [12]. Loss-of-function mutations in either *ABCG5* or *ABCG8* have been identified as a cause of sitosterolemia, a rare autosomal recessive disorder characterized by elevated plasma levels of plant sterols due to increased intestinal absorption of dietary sitosterol and decreased biliary sterol secretion [13, 14]. The majority of patients with sitosterolemia are characterized by normal to moderately elevated plasma cholesterol concentrations [15, 16]. On the other hand, the common *ABCG8*: c.55G > C polymorphism (rs11887534) has been reported to account for variability in plasma concentrations of: triglycerides, total cholesterol concentrations, LDL-C concentrations and HDL-C concentrations [14, 17–20] as well as for the variability in plasma lipid parameters in response to treatment with statins [7, 14, 21]. However, other authors have not confirmed associations of *ABCG8*: rs11887534 with plasma lipid levels [22–24]. In addition, till now only few studies on *ABCG8*: rs11887534 have been conducted with Slavic populations [22, 25]. Therefore, we decided to analyze both baseline plasma lipids and changes in their concentrations in response to statin treatment in regard to the *ABCG8*: rs11887534 polymorphism in Polish Caucasian patients.

## Materials and methods

The study group consisted of 170 consecutive adults (52 males and 118 females, aged from 38- to 84-years old) recruited in an outpatient clinic in Szczecin according to the protocol described previously [26]. All recruited participants were Caucasian patients of Polish origin living in Szczecin, the largest city in West Pomerania. Inclusion criteria were as follows: age > 18 years old, the presence of a lipid disorder and treatment either with atorvastatin (10–20 mg per day) or with simvastatin (20–40 mg per day) for a minimum of 2 months. Exclusion criteria were: smoking, thyroid disease (hyperthyroidism or hypothyroidism), or if, after extensive interview, patients had not complied fully with instructions, including a diet low in fat. Clinical data from patients’ records included: age, gender, body mass index (BMI) calculated as (body mass, kg)/(height, m^2^), duration of statin treatment, the daily dose of statin, and the presence of arterial hypertension, diabetes mellitus or coronary artery disease (Table 1). Laboratory data from patients’ records included: serum concentrations of triglycerides (TG), total cholesterol (TC), LDL-cholesterol (LDL-C) and HDL-cholesterol (HDL-C). Lipid concentrations were measured before (time 1) and after (time 2) the statin treatments as described previously [26]. In addition, absolute (∆_2–1_ = time 2 − time 1) or relative (∆_%_ = 100*(time 2 − time 1)/time 1) differences were calculated for TG, TC, LDL-C and HDL-C (Table 2). Peripheral blood samples (5 ml) were drawn before statin treatment and stored at − 20 °C until DNA isolation. All patients gave informed, written consent to participate in the study, which was approved by the bioethics committee at the Pomeranian Medical University, Szczecin, Poland. Genomic DNA was extracted from peripheral blood leukocytes using a commercially available DNA isolation kit (QIAamp Blood DNA Mini Kit, QIAGEN, Germany). Each DNA sample was used as a template for PCR to amplify a 130-bp *ABCG8* sequence, including rs11887534. PCR was performed using: 5′–GCTGGGTCTAAGAGAGCTGC–3′ as the forward primer and 5′–CTTCCCATTGCTCACTCACC–3′ as the reverse primer. Subsequently, the PCR amplification products were purified using Exonuclease I and FastAP Thermosensitive Alkaline Phosphatase (ThermoFisher Scientific Inc., Waltham, MA USA) according to manufacturer procedures. The purified amplicons were subjected to a mini-sequencing reaction using an ABI PRISM SNaPshot Multiplex Kit (Applied Biosystems) with extension primer 5′–GACTGACTGACTGACTGACTGACTGACTGACTGACTTGCTCACTCACCGAGGTAT–3′. Capillary electrophoresis of the mini-sequencing products was performed on an ABI PRISM 3100-Avant genetic analyzer (Applied Biosystems). The mini-sequencing products were visualized and analyzed with GeneMapper™ v4.1 Software (Applied Biosystems). All DNA samples were genotyped using a blind method, i.e. the samples were anonymously labeled by one person and then genotyped by the second person.

**Table 1:** Basic characteristics of patients in regard to their *ABCG8* genotype

Variable	All	*ABCG8* genotype		*p*
	(*n* = 170)	GG (*n* = 147)	GC + CC (*n* = 22 + 1)	GC + CC vs GG
Males, *n* (%)	52 (30.6)	47 (32.0)	5 (21.7)	0.455
Age (years)	66 (57, 73)	66 (56,73)	68 (59, 73)	0.408
BMI (kg/m^2^)	27.3 (24.4, 30.4)	27.2 (24.4, 30.4)	28.0 (24.2, 31.9)	0.684
Arterial hypertension, *n* (%)	134 (78.8)	118 (80.3)	16 (69.6)	0.372
Diabetes mellitus, *n* (%)	49 (28.8)	41 (27.9)	8 (34.8)	0.584
Coronary artery disease, *n* (%)	37 (21.8)	33 (22.4)	4 (17.4)	0.667
Duration of treatment with statin (months)	9 (4, 20)	9 (4, 19)	12 (5, 24)	0.630
Simvastatin equivalent dose (mg/day)	40 (20–40)	40 (20–40)	40 (20–40)	0.849
Patients using higher (≥ 40 mg/day) simvastatin equivalent dose, *n* (%)	85 (50.0)	73 (50.0)	12 (52.2)	1.000

Quantitative data are presented as median (lower quartile, upper quartile)

**Table 2:** Lipid parameters of the patients in regard to their *ABCG8* genotype

Variable	Time code	All	*ABCG8* genotype		*p*
		(*n* = 170)	GG (*n* = 147)	GC + CC (*n* = 22 + 1)	GC + CC vs GG
TG	1	1.57 (1.26, 2.18)	1.59 (1.28, 2.29)	1.46 (1.11, 1.81)	0.089
	2	1.26 (0.97, 1.77)	1.25 (0.97, 1.84)	1.28 (0.87, 1.55)	0.571
	∆_2–1_	− 0.34 (− 0.82, 0.02)	− 0.36 (− 0.94, 0.01)	− 0.02 (− 0.65, 0.15)	0.183
	∆_%_	− 24.5 (− 46.3, 2.1)	− 25.4 (− 47.4, 0.6)	− 2.0 (− 39.7, 9.6)	0.234
TC	1	6.66 (6.01, 7.38)	6.73 (6.06, 7.36)	6.37 (5.26, 7.59)	0.238
	2	4.51 (4.01, 5.13)	4.53 (4.04, 5.15)	4.27 (3.76, 4.92)	0.294
	∆_2–1_	− 2.10 (− 2.88, − 1.37)	− 2.12 (− 2.77, − 1.37)	− 1.94 (− 2.64, − 1.45)	0.680
	∆_%_	− 31.0 (− 40.2, − 22.1)	− 31.1 (− 40.2, − 22.0)	− 31.0 (− 41.1, − 23.6)	0.973
LDL-C	1	4.36 (3.66, 5.04)	4.37 (3.68, 5.04)	4.08 (3.43, 4.65)	0.330
	2	2.42 (2.00, 2.95)	2.41 (1.97, 2.98)	2.55 (2.01, 2.92)	0.911
	∆_2–1_	− 1.86 (− 2.59, − 1.09)	− 1.92 (− 2.59, − 1.21)	− 1.47 (− 2.59, − 0.94)	0.272
	∆_%_	− 44.2 (− 53.4, − 29.4)	− 44.3 (− 53.6, − 30.3)	− 34.9 (− 51.3, − 25.0)	0.432
HDL-C	1	1.45 (1.42, 1.66)	1.45 (1.22, 1.63)	1.45 (1.24, 1.92)	0.466
	2	1.42 (1.22, 1.66)	1.45 (1.22, 1.71)	1.27 (1.11, 1.53)	0.028
	∆_2–1_	0.00 (− 0.23, 0.21)	2.00 (− 7.0, 0.21)	− 0.13 (− 0.44, − 0.02)	0.003
	∆_%_	0.0 (− 14.6, 14.3)	3.1 (− 12.9, 15.9)	− 9.8 (− 25.3, − 1.8)	0.002

Lipids concentrations and absolute differences in lipid concentrations are measured in millimols per liter (mmol/l). The relative differences in lipid concentrations are expressed as a percentage. Data are presented as median (lower quartile, upper quartile)

Normal distribution of quantitative data was tested using Shapiro–Wilk tests. Since the majority of quantitative variables were not normally distributed, we presented all of them as median values with lower (Q1) and upper quartiles (Q3). Quantitative data were compared between genotype groups using Mann–Whitney tests. Categorical data and the divergence of *ABCG8* genotype frequencies from Hardy–Weinberg equilibrium were assessed using chi-squared tests. Statistical significance was defined as *p* < 0.05. We calculated the statistical power of the study to detect significant differences of relative changes (Δ%) in lipid parameters during statin treatment between genotype groups, assuming that standard deviations of the changes were equal to 15% for TC, 20% for LDL-C, 25% for HDL-C and 50% for TG. The power with 170 subjects and a minor allele frequency of 7% was sufficient to detect with 80% probability true differences equivalent to 10% for TC, 13% for LDL-C, 16% for HDL-C and 32% for TG, between genotype groups using a dominant model. All data were analyzed using a data analysis software system (Dell Statistica, version 13. Dell Inc. 2016, software.dell.com).

## Results

There were 147 GG homozygotes (86.5%), 22 GC heterozygotes (12.7%) and one CC homozygote (0.6%) in the studied group, and *ABCG8*: rs11887534 genotype distribution conformed to expected Hardy–Weinberg equilibrium (*p* = 0.582). The frequency of the minor *ABCG8*: c.55C allele was 7.1%. No significant differences in gender composition, age, BMI, prevalence of arterial hypertension, frequency of diabetes mellitus, prevalence of coronary artery disease, duration of treatment with statin and frequency of patients treated with a higher (≥ 40 mg/day) simvastatin equivalent dose were found between subjects homozygous for the wild-type *ABCG8* allele (c.55G) and individuals having at least one mutated allele (GC or CC genotype) (Table 1).

There were also no significant differences in regard to *ABCG8* variants for baseline TG, TC, LDL-C and HDL-C concentrations, for TG, TC or LDL-C concentrations after treatment with statin as well as for absolute and relative differences in TG, TC or LDL-C levels. The only significant differences between both genotype groups of patients concerned HDL-C concentrations after statin use as well as absolute and relative changes in HDL-C concentrations. In contrast to GG homozygotes, patients with GC + CC genotypes showed a decrease in post-statin HDL-C concentrations, and negative absolute and relative differences in HDL-C concentrations (Table 2).

## Discussion

The *ABCG8 locus* is one of many loci identified to be associated with blood lipid levels [27–29]. The c.55G > C transversion (rs11887534) in the *ABCG8* gene causes the substitution of aspartic acid (Asp, D) by histidine (His, H) at amino acid position 19 (p.Asp19His) of the sterolin-2. An aspartic acid at amino acid position 19 is highly conserved from plants to vertebrates and its substitution by histidine results in the loss of negative charge [14]. Therefore, it has been speculated that this conformational change might increase the function of the ABCG5/ABCG8 transporter [19]. However, there are no experimental reports confirming the influence of rs11887534 on the expression or activity of this transporter so far [21, 30].

The frequency of the minor *ABCG8*: c.55C allele of 7.1% in our patients was very similar to its frequency previously reported by Krawczyk et al. in Poles (7.5%) [25] or by Hubacek in Czechs, who are also of Slavic origin (6.7%)[22]. The prevalence of rs11887534 in Polish subjects was also close to its frequencies in other European populations, which ranged from 5.4 to 10.6% [10, 24, 31].

There was no significant association between *ABCG8*: rs11887534 polymorphism and plasma concentrations of triglycerides, total cholesterol, LDL-cholesterol and HDL-cholesterol in our studied patients. The lack of such associations has also been reported not only in Czech patients [22], but also in healthy white subjects of non-Hispanic origin in the Dallas metropolitan area [23], in a cohort of Indian patients with coronary artery disease [21] and in a large cohort of Dutch patients with heterozygous familial hypercholesterolemia [24]. However, the results of a study by Kajinami et al. carried out in 338 multi-ethnic patients in the USA revealed that plasma cholesterol concentrations in subjects carrying at least one minor *ABCG8*: c.55C allele were significantly lower than in wild-type homozygotes [14]. Gylling et al. reported that in mildly hypercholesterolemic Finns the minor *ABCG8*: rs11887534 variant was associated not only with lower total cholesterol but also with lower LDL cholesterol [18]. In addition, Acalovschi et al. have found that both plasma cholesterol and plasma triglycerides were lower in Romanian patients carrying at least one mutated rs11887534 allele as compared to wild-type homozygous subjects [17]. In turn, Junyent et al. revealed that the participants of the Boston Puerto Rican Health Study carrying the minor rs11887534 allele displayed lower concentrations of HDL-C only if they were smokers [20]. In contrast to these aforementioned results, Chen et al. showed that the *ABCG8*: c.55C allele in Taiwanese subjects consuming an ordinary Chinese diet (a diet with lower cholesterol and higher phytosterol content compared to a Western diet) was associated with both higher total cholesterol and higher LDL-cholesterol [19].

Reports concerning the efficacy of statin treatment in regard to *ABCG8*: rs11887534 polymorphism are scarce [7, 14, 21]. In 2004 Kajinami et al. revealed that post-atorvastatin TC and post-atorvastatin LDL-C were significantly lower and adjusted percent reductions of LDL-C concentrations were significantly greater in subjects carrying at least one minor *ABCG8*: rs11887534 allele (c.55C) as compared to GG homozygotes [14]. Srivastava et al. reported that post-treatment TC was significantly lower, and percent reduction of LDL-C was significantly greater, in Indian patients with coronary artery disease having at least one *ABCG8*: c.55C allele than in subjects with GG homozygous genotype. However, the *ABCG8*: rs1887534 polymorphism in these patients was not independently associated with both absolute or a percent reduction in LDL-C in stepwise multiple regression analysis including: age, gender, pretreatment lipid levels and *ABCG8* genotype as independent variables [21]. On the other hand, Chien et al. revealed a significant association of an *ABCG8* haplotype including wild-type rs11887534 with reduction in LDL-C after statin treatment in a Chinese population [7].

In contrast to above reports, we have found no association of *ABCG8*: rs11887534 polymorphism with response of LDL-C, total cholesterol and triglyceride levels to statin treatment. However, we have revealed a significant decrease in HDL cholesterol after statin use in our patients carrying at least one minor c.55C allele as compared with wild-type *ABCG8* homozygotes. Ethnic-dependent differences, both in the frequency of *ABCG8* polymorphism and in the prevalence of environmental factors (e.g. dietary habits), should be taken into consideration as major reasons for the inconsistency of results among studies.

Treatment with statin usually moderately increases the serum concentration of HDL cholesterol in a majority of patients but some patients experience a paradoxical decrease in HDL-C levels after such pharmacotherapy [32]. In addition, Ota et al. suggested that a paradoxical decrease in HDL cholesterol after statin treatment might be an independent predictor for long-term adverse cardiovascular events in patients with acute myocardial infarction [32]. Hasvold et al. noted a decrease in HDL-C of > 0.1 mmol/l in 20% of patients treated with statins (96% of the cohort were initiated on simvastatin with a mean dose of 20 mg/day), and the group of patients with reduction in HDL-C comprised more women, had a higher HDL-C at baseline (1.69 mmol/L) and less diabetes compared with the unchanged HDL-C group [5]. In our study more than 34% of patients (58 out of 170 subjects) experienced an HDL-C lowering of > 0.1 mmol/l, but this phenomenon was not associated with gender, baseline HDL-C or the prevalence of diabetes. As our study was based on data from patients’ records in a primary care clinic, perhaps some unmeasured or unregistered confounders may have influenced our results. Hasvold et al. have also suggested that low compliance to statin treatment should be taken into consideration as a potential explanation of paradoxical decreases in serum HDL cholesterol [5]. We observed no significant difference in frequency of the reduction of LDL cholesterol > 0.5 mmol/l between GC + CC patients and GG wild-type homozygotes (95.6% and 89.1%, respectively). In addition, no significant differences between both aforementioned groups were found in duration of treatment with statin, in simvastatin equivalent dose or in the frequency of patients using higher (≥ 40 mg/day) simvastatin equivalent dose. Therefore, both low compliance to use of statin or low adequacy of treatment with statin should be rather excluded as the reasons for the paradoxical HDL-C decreases. On the other hand, despite the crucial role of the ABCG5/ABCG8 transporter in the induction of reverse cholesterol transport [33], the mechanism underlying the reduction in HDL cholesterol in *ABCG8*: c.55C carriers treated with statins remains unclear. Junyent et al. have suggested that the rs3806471 polymorphism, with a change located in the binding motif for FXR (Farnesoid X Receptor) in the *ABCG8* promoter, might modulate the expression of this gene [20]. In addition, Habeos revealed that simvastatin decreases the expression of FXR at both the RNA and protein levels and down-regulates its DNA-binding activity [34]. However, there is no strong linkage disequilibrium between rs3806471 and rs11887534 in European subjects [35].

Limitations of the study are the study design and the fairly low sample size. In contrast to previous studies by Kajinami et al. [14], Srivastava et al. [21] or Chien et al. [7] we carried out a retrospective observational study in 170 subjects whose data (except *ABCG8* genotypes) were obtained from available medical records of a single-outpatient clinic. The majority of patients with dyslipidaemia in this clinic were treated either with simvastatin or with atorvastatin, and other statins (e.g. rosuvastatin) or cholesterol absorption inhibitor (ezetimibe) were used to a marginal extent. Therefore, we are fully aware that our results should be interpreted with caution and need to be replicated using a larger sample size of patients qualified in randomized manner to the groups treated with CYP3A4-metabolized statins (simvastatin or atorvastatin), non-CYP3A4-metabolized statins (fluvastatin, pravastatin or rosuvastatin) or ezetimibe. Ezetimibe, a selective cholesterol absorption inhibitor blocking Niemann-Pick C1 like-1 (NPC1L1) protein, is effective in sitosterolemia caused by *ABCG5/ABCG8* mutations but Caamano et al. reported no association between *ABCG8*: rs11887534 polymorphism and lipid response (including HDL changes) to treatment with ezetimibe [36]. On the other hand, Zsiros et al. revealed that *NPC1L1*: c.-133A > G polymorphism modifies the ApoA1 response to ezetimibe and therefore, rather than altering HDL concentration, may alter the effects of ezetimibe on the structure and function of HDL particles [37].

In conclusion, our results suggest that the c.55C allele of *ABCG8*: rs11887534 polymorphism might be associated with paradoxical decrease in HDL cholesterol levels in response to the statin treatment of Polish patients.
